# An Account of Two Cases of Polydipsia, or Excessive Thirft

**Published:** 1792

**Authors:** 


### VIII. *An Account of two Cafes of Polydipfia, or exceffive Thirft.*

ALMOST all the modern nofologifts have introduced into their fyftems a difeafe to which, on account of the exceffive thirft that forms its characteriftic fymptom, they give the name of Polydipfia; but in none of the examples of it they have been able to collect does it appear to have been idiopathic; and Dr. Cullen expreffly fays that it is almoft* always fymptomatic. A very curious inftance, however, of fuch an affection, apparently depending on a peculiarity of temperament, or what is called *idiofyncracy*, occurs in a woman now living at Paris, of whofe cafe the firft account was given, by M. Beffejon de la Chaffagne, in

* The words "polydipfia *fere* femper fymptomatica eft," ufed by Dr. Cullen, might lead the reader to imagine that he had *fometimes* feen it exift as an idiopathic difeafe; but the varieties he mentions of it, viz. Polydipfia febrilis, polydipfia hydropica, polydipfia fluxuum, and polydipfia a venenis, are all of them collected from M. Sauvages as inftances of fymptomatic affection: he gives no example of it from his own experience.—See Synopf. Nofol. Meth. 8vo. Edin. 1785. Tom. II. p. 320.

a letter a letter which we shall here translate from the Paris Journal of May 1, 1789.

" *To the Authors of the Journal* *.

" Paris, April 18th, 1789.

" Gentlemen,

" You will interest equally the humanity and " curiosity of the Public, by inserting in your " Journal

* " *Aux Auteurs du Journal.*

" A Paris, ce 18 Avril, 1789.

" Messieurs,

" Vous intereſſerez également l'humanité & la curioſité, " en inſerant dans votre Journal l'anecdote ſuivante qui pa- " roîtra, ſans doute, un phénomène. Je me ſuis aſſuré avec " l'exactitude la plus ſcrupuleuſe des faits que j'annonce ici; " mais je laiſſe aux lecteurs d'en expliquer la nature & la " cauſe.

" Catherine Bonfergent a été remarquée dès l'age le plus " tendre. Une ſoif brûlante, une alteration ſans exemple, " dont elle eſt continuellement tourmentée depuis ſa naiſſance, " ont toujours fixé ſur elle l'attention des obſervateurs. Ses " parens, après en avoir confié les premiers ſoins à une nour- " rice, la retirerent auprès d'eux à ſa troiſième année. Ils " trouvèrent bientôt extraordinaire la conſommation d'eau " qui ſe faiſoit dans leur maiſon depuis quelque tems, lorſ- " qu'enfin ils s'appercurent que leur enfant en buvoit près " de deux ſeaux chaque jour. Ils attribuèrent d'abord à une " mauvaiſe éducation ce qui étoit en effet chez elle un beſoin " ſurprenant,

" Journal the following anecdote, which, with-

" out doubt, will be confidered as a phenome-

" non. I have affured myfelf, with the moft

" fcrupulous accuracy, of the facts which I

" announce, but I leave to the reader to ex-

" plain their nature and caufe.

" Catherine Bonfergent has been remarked
" from her tendereft years. A burning thirft,
" a drought without example, with which fhe
" has

" furprenant, mais naturel. En vain ils ont voulu la corri-
" ger de ce defaut par des careffes ou des menaces, en lui re-
" fufant ou lui diminuant la quantité d'eau qu'elle buvoit;
" ils ont été bien plus furpris encore de la voir chercher fe-
" crètement tous les moyens de pouvoir fe fatisfaire; en été
" avec la premiere eau qu'elle pouvoit fe procurer, en hiver
" avec de la neige & des glacons, & toujours elle avoit foin
" de fe réferver de quoi boire abondamment pendant la nuit.

" Les mauvais traitemens que ce befoin lui attiroit de fes
" parens l'ont obligée de les quitter. Elle eft venue fervir à
" Paris chez des maitres plus indulgens à qui elle n'a pu fe
" cacher; mais fa bonne conduite a merité d'eux de n'en re-
" cevoir aucuns reproches.

" A l'age de 22 ans elle s'eft mariée avec le nommé Fery,
" favetier, à qui elle a déguifé fon défaut pour l'époufer.
" Elle a eu de lui huit enfans; il lui en refte trois, & elle eft
" enceinte d'un neuvième. Ce qui paroit furtout extraordi-
" naire, c'eft qu'au moment de fes couches, au lieu d'ufer
" d'alimens & de liqueurs qui fembleroient plutôt devoir la
" fortifier,

" has been continually afflicted from the time
" of her birth, have always fixed on her the
" attention of perfons of obfervation. Her
" parents, after having entrufted the firft care
" of her to a nurfe, took her home when fhe
" was three years old. It was not long before
" they obferved that an extraordinary quantity
" of water was confumed in the houfe, and at

" fortifier, elle préfère pour contenter fa foif, qui eft alors

" plus brûlante, de boire, prefque fans interruption, trois on

" quatre pintes d'eau la plus fraiche. Dans les rigueurs de

" l'hiver dernier, cette femme enceinte a bu jufqu'à deux

" voies d'eau en 24 heures; & fon mari, ne pouvant fournir

" à cette depenfe, étoit obligé de ramaffer de la neige & des

" glacons qu'il faifoit fondre. L'eau, qui s'eft vendue fix

" fols la voie, lui coûtoit plus qu'il ne gagnoit de fon tra-

" vail.

" Cette femme n'a jamais fait ufage d'aucunes fortes de

" liqueurs fortes, & s'il lui arrivoit feulement de boire un

" verre de vin, elle éprouveroit un faififfement dans tous fes

" membres, & on croiroit la voir tomber en fincope; d'ail-

" leurs elle n'eft pas hidropique, elle jouit même d'une affez

" bonne fanté, elle rend naturellement toute l'eau qu'elle

" boit; mais ce qui paroît furprenant encore, c'eft que cette

" eau eft d'une fétidité extraordinaire. Cette femme de-

" meure Hôtel des Arts, F. St. Martin.

" Signé BESSEJON DE LA CHASSAGNE,

" Prétre de St. Laurent."—Journal de Paris, 1 Mai, 1789.

" length

" length they difcovered that their daughter " drank, every day, to the amount of nearly " two pailfuls. At firft they attributed to im- " proper education what in reality was the " effect of a furprifing, though natural, appe- " tite. It was to no purpofe that they at- " tempted to correct this defect by careffes or " threats, by denying her water, or leffening " the quantity of what fhe drank; and they " were ftill more furprifed to fee her fecretly " availing herfelf of every means to fatisfy " her thirft. In fummer fhe drank the firft " water fhe could meet with, in winter fhe had " recourfe to fnow and ice, and fhe was always " careful to referve as much as would enable " her to drink abundantly during the night.

" The ill treatment this difpofition occa- " fioned her to experience from her parents, " at length, obliged her to quit them; and fhe " came to Paris and lived as a fervant with dif- " ferent families, who were more indulgent to " her; for although the infirmity in queftion " was not to be concealed, her good conduct " in other refpects fecured her from reproach.

" At the age of twenty-two years fhe mar- " ried one Fery, a cobler, from whom fhe con- " trived to conceal her complaint till after

" their

" their marriage. She has had by him eight " children, three of whom are ſtill alive, and " ſhe is now pregnant with a ninth. What " appears particularly extraordinary is, that, " during her lyings-in, inſtead of having re- " courſe to ſuch food and liquors as would " ſeem to be moſt likely to ſtrengthen her, ſhe " chuſes rather, for the ſake of ſatisfying her " thirſt, which at thoſe times is more intenſe, " to drink, almoſt without interruption, three " or four quarts of the coldeſt water. Du- " ring the ſevere cold of laſt winter this wo- " man, who was then pregnant, drank to the " amount of four pailfuls of water in twenty- " four hours; and her huſband being unable to " afford the expence of ſuch a conſumption, " was under the neceſſity of ſupplying her with " melted ſnow and ice. The price of a load " (two pailfuls) of water, at that time, was " ſix *ſols*; and the quantity ſhe required would " have coſt him more than he earned by his " work.

" This woman has never made uſe of any " ſort of ſtrong liquors, and if ſhe drinks " only a ſingle glaſs of wine ſhe feels an un- " eaſy ſenſation in all her limbs, and ſeems to " be in danger of fainting. She is not drop-

" ſical;

" fical; ſhe even enjoys a pretty good ſtate of

" health; ſhe voids naturally all the water ſhe

" drinks, but her urine is extraordinarily fœ-

" tid. She lives at the Hotel des Arts, Faux-

" bourg Saint Martin.

" Signed BESSEJON DE LA CHASSAGNE,

" Prêtre de St. Laurent."

The facts related by M. de la Chaſſagne ſeemed, if they might be relied on, to afford an inſtance of an affection, at any rate extremely rare, if not altogether without example; but their value, like that of every other fact, depended on their authenticity. There was a poſſibility that the writer of the account, without any intention of ſtating more than was true, might have been deceived by the patient or her friends, and that the ſtory in queſtion might, on farther inquiry, like too many other extraordinary aſſertions, be found to be greatly exaggerated, or even to have its origin in ignorance or impoſture. A narrative ſo remarkable ſeemed, however, to be deſerving of inveſtigation, and accordingly the Editor of this work ventured to direct the attention of ſome of his medical friends at Paris to the caſe, and to ſolicit licit their aſſiſtance in aſcertaining the degree of confidence it might merit.

The firſt communication he was favoured with on this ſubject was from M. Tenon, Profeſſor of Anatomy, and Member of the Royal Academies of Sciences and Surgery at Paris, who, in a letter, dated Paris, September 7th, 1790, ſays, "This woman*, Fery, at the "Hotel des Arts, Fauxbourg St. Martin, is "now thirty-nine years old, and pregnant of

* "Cette femme, Fery, Hotel des Arts, rue du Fauxbourg "St. Martin, maintenant en Septembre, 1790, agée de 39 "ans, eſt enceinte de ſon dixieme enfant. Selon elle, de- "puis l'age de quatre a cinq ans juſqu'a celui de ſeize a dix "huit, elle buvoit un ſeau d'eau, c'eſt a dire, dix pintes pe- "ſant chacune deux livres de ſeize onces par jour; depuis "cette epoque elle en a bu conſtamment deux ſeaux & quel- "que fois trois par jour de vingt quatres heures. A chaque "coup elle boit un peu plus d'une pinte. Sa ſanté n'en pa- "roit point alterée; ſeulement elle reſſent un peu de chaleur "a la levre inferieure, & qui en même temps la durcit. A "la moindre indiſpoſition la ſoif diminue.

"Cette femme n'a conſervé que deux enfans. Le plus agé "a ſeulement huit a dix ans. Ils ne partagent point l'in- "commodité de leur mere.

"Cette femme enfin eſt de moyenne ſtature; maigre; & "blonde tirant ſur le roux. J'ai fait prendre ces renſeigne- "mens par une perſonne ſure; on les tient de la malade elle "même; mais je ne garantis pas qu'elle n'en ait impoſé."

"her

" her tenth child. According to her own ac-

" count, from the age of four or five years, to

" that of ſixteen or eighteen, ſhe drank one of

" our pailfuls of water, that is to ſay, ten

" quarts, (or Paris pints) each weighing two

" pounds of ſixteen ounces, daily. Since that

" period ſhe has conſtantly drank twenty quarts,

" and ſometimes thirty, in the ſpace of twen-

" ty-four hours. Every time ſhe drinks ſhe

" ſwallows rather more than a quart. Her

" health does not appear to be affected, only

" ſhe experiences a little heat in her under lip,

" which at the ſame time hardens it. When

" ſhe is in the leaſt indiſpoſed her thirſt dimi-

" niſhes.

" She has reared only two children: the
" eldeſt is not more than eight or ten years
" old. Neither of them partake of their mo-
" ther's complaint.

" This woman is of a middle ſtature;
" lean; and of a fair complexion, inclining
" to red.

" I have employed a perſon on whom I can

" depend to procure for me theſe particulars,

" and he has them from the patient herſelf;

"but I cannot be ſure that ſhe has not impoſed "on him *."

The next account the Editor received of this caſe was from Mr. William Maiden, of Stroud in Kent, an ingenious ſtudent of phyſic, who

* To the information here given, relative to the woman in queſtion, M. Tenon adds the following inſtance of Polydipſia from his own experience: — "This fact," ſays he, "reminds me of another that has occurred to me in the courſe "of my practice. An Advocate, who was eagerly engaged "in hunting, and much heated, happened to fall into a rivulet. To this accident ſucceeded a thirſt, which induced "him to drink from eight to ten quarts daily. At the end "of three months, during which this complaint laſted, he "came to Paris, and was cured chiefly by the uſe of a chalybeate water.

"There is another inſtance of exceſſive thirſt occaſioned "by antimony. This laſt fact is by Wepfer.—See the *Collection Academique*, Tome VII. page 584." — "Ce fait "m'en rappelle un autre qui s'eſt preſenté a moi dans le cours "de ma pratique. Un Avocat, entrainé par la chaſſe, & "etant en ſueur, tomba dans un ruiſſeau. A cet evenement "ſucceda une ſoif qui le portoit a boire environ huit a dix "pintes par jour. Au bout de trois mois que duroit cet etat "il vint a Paris, & fut gueri par l'uſage de l'eau ferrugineuſe.

"On a un autre exemple de ſoif extraordinaire cauſée par "l'antimoine. Cette derniere obſervation eſt de Wepfer; "*Collection Academique*, Tom. VII. page 584."

went went lately from London to Paris, and who, at his requeſt, viſited this woman in Auguſt, 1791. Mr. Maiden found her rather thin in the face, but ſeemingly in good general health, without any ſwelling of the legs or preternatural enlargement of the belly; and ſhe related to him nearly the ſame circumſtances of her caſe that the reader has ſeen mentioned in the preceding accounts. But Mr. Maiden, who delayed this inquiry till the day before he intended to leave Paris, having been able to remain with her only a few minutes, the truth of the facts ſtill reſted chiefly on the aſſertion of the woman herſelf, and nothing leſs than the ocular teſtimony of ſome intelligent perſon, whoſe accuracy might be fully relied on, ſeemed ſufficient to eſtabliſh their authenticity. Such a teſtimony has, at length, been obtained through the obliging exertions of M. Souville, Phyſician at Calais, and M. Parmentier, Apothecary Major of the Royal Hoſpital of Invalids at Paris, at whoſe requeſt M. Brougniart, who is known to the Editor of this work as a very accurate and ingenious ſtudent of phyſic, readily undertook the inveſtigation of the caſe. The following papers relative to this ſubject, with which we ſhall cloſe our account of the caſe, will ſhow the ſatisfactory manner in which the facts in queſtion have been aſcertained:

1. *Extract of a Letter from M.* Parmentier, *Apothecary Major of the Royal Hoſpital of Invalids,* &c., *to M.* Souville, *Phyſician at Calais, and Member of the Royal Medical Society at Paris,* &c., *dated Paris, October 27th,* 1791.

" I have delayed, Sir, doing myſelf the ho-

" nour of anſwering your letter till I ſhould

" be furniſhed with the particulars of the caſe

" Dr. Simmons has requeſted of us; and I

" thought I could not do better than to re-

" queſt M. Brougniart to procure them for

" him. You will judge from the incloſed pa-

" pers how well he has acquitted himſelf of a

" commiſſion which I myſelf ſhould have rea-

" dily undertaken, had I not had reaſon to

" hope that it would be ſtill more completely

" executed by phyſicians who, though young,

" are good obſervers *."

2. *Ex-*

* " J'ai attendû, Monſieur, pour avoir l'honneur de vous

" repondre que je fuſſe muni de l'obſervation que le Docteur

" Simmons nous a demandé. Je n'ai crû pouvoir mieux faire

" que de charger M. Brougniart de lui la procurer. Vous

" jugerez

2. *Extract of a Letter from M.* Brougniart, *Student of Physic at the Royal Hospital of Invalids at Paris, to Dr.* Simmons, *dated Paris, October* 25, 1791.

" You * wrote lately, Sir, to M. Souville,

" for the purpose of procuring some authentic

" information relative to a woman at Paris

" who drinks a great quantity of water. You

" expressed to him a wish that some person,

" whose accuracy could be relied on, might be

" engaged to visit this woman, and see with

" his own eyes the truth of this fact. M. Sou-

" ville

" jugerez par les pieces c'y jointes s'il s'est bien acquitté de la

" commission que j'aurois volontiers fait moi même, sans l'es-

" perance ou j'etois quelle ferait encore plus completement

" remplie par des medecins qui, quoique jeunes, voient bien."

* " Vous ecrivites, Monsieur, dernièrement a M. Sou-

" ville pour avoir des renseignemens exacts sur une femme

" de Paris qui buvoit une tres grande quantité d'eau. Vous

" lui disiez que vous desiriez que quelque personne sure voula

" suivre cette femme pendant assez de temps pour voir par ses

" propres yeux la verité de ce fait. M. Souville ecrivit a M.

" Parmentier & lui envoya votre lettre. M. Parmentier,

" avec lequel j'ai le bonheur d'être extremement lié, sachant

" combien je desirois trouver l'occasion de vous marquer ma

" reconnoissance des bontés que vous avez eû pour moi pen-

" dant

"ville wrote to M. Parmentier, and fent him "your letter. M. Parmentier, with whom I "have the good fortune to be intimately con- "nected, knowing how much I wifhed for an "opportunity of making you fome return for "your kindnefs to me during my ftay in Eng- "land, did me the favour to put into my hands "your letter, and at the fame time engaged "me to make the inquiries you defired.

"Being perfuaded that whenever the object "is to afcertain a fact two perfons obferve "better than one, and muft neceffarily infpire "more confidence, I communicated your letter "to a fociety who meet for fcientific purpofes, "and of which I am a member. The fociety "joined with me a young phyfician for the

"dant mon fejour en Angleterre, voulu bien me remettre "votre lettre & me charger de prendre les renfeignemens "que vous demandiez.

"Perfuadé que lorfqu'il s'agit de conftater un fait deux "perfonnes voyent mieux qu'une & doivent infpirer plus de "confiançe, je communiquai votre lettre a une Société qui "s'occupe des Sciences & a la quelle je fuis affocié ; on "m'adjoignit un jeune medecin, & nous obfervames enfem- "ble, chez moi, la femme en queftion. Plufieurs autres "membres de la même Société furent prefens aux obfervations "ainfi que vous pouvez le voir par le rapport ci joint que "j'ai l'honneur de vous envoyer."

" purpofe of affifting me in the inquiry, and
" we faw together, in my apartments, the wo-
" man in queftion. Several other members
" of the fame fociety were prefent during the
" inveftigation, as you will fee by the annexed
" Report, which I have the honour to fend to
" you."

3. *Report* made to the Philomatical Society, relative to a Woman who drinks a great Quantity of Water; by M. M.* Bellot *and* Brougniart. *Read at a Meeting of the Society on Saturday the 22d of October,* 1791.

" The Philomatical Society, being defirous
" of complying with the requeft made by M.
" Parmentier,

* *Rapport fait à la Société Philomatique, fur une Femme qui boit une grande Quantité d'Eau; par M. M.* Bellot & Brougniart. *Seance du Samedi, 22 Octobre,* 1791.

" La Société Philomatique, défirant repondre à la demande
" qui lui a été faite par M. Parmentier au nom du Docteur
" Simmons, nous a nommé pour examiner le tempérament
" & les habitudes d'une femme qui boit beaucoup d'eau.

" Nous nous fommes tranfportés en confequence, Samedi,

" 15 Octobre, 1791, au Fauxbourg St. Martin, Hotel des

" Arts, chez la femme en queftion. Ne l'ayant point ren-

" contré chez elle, nous allames à la place où travailloit fon

" mari,

" Parmentier, in the name of Dr. Simmons,
" appointed us to examine the temperament
" and habits of a woman who drinks a great
" quantity of water.

" We

" mari, après avoir pris auparavant quelques informations

" auprès du Portier de la Maiſon, qui furent conformes à ce

" que l'on nous avoit deja dit. Nous trouvames cette femme

" avec une cruche d'eau à côté d'elle. Nous primes jour, &

" il fut convenu, qu'elle viendroit paſſer une journée entière

" chez l'un de nous.

" Nous nous reunimes, en effet, Lundi, 17 Octobre, 1791,

" & reçûmes de cette femme les renſeignemens ſuivans.

" Catherine Bonfergent, epouſe de Jacques Fery, ſavetier,

" demeurant à Paris, Hôtel des Arts, Fauxbourg St. Martin,

" paroiſſe St. Laurent, eſt agée de 40 ans. Elle eſt née à

" Senlis.

" Elle eſt très blonde ; ſa peau eſt fine & marquée de

" taches de rouſſeur. Elle eſt plus maigre que graſſe, & pa-

" roit être d'un temperament bilieux. Ses bras ſont plus

" maigres que le reſte de ſon corps.

" Elle fut miſe en ſevrage chez ſa grande mère, qui, bu-

" vant beaucoup de vin, lui en fit boire auſſi. De retour

" chez ſa mère, elle vomiſſoit tout ce qu'elle prenoit. Les

" matières qu'elle vomiſſoit étoient noires.

" Dès ſa plus tendre jeuneſſe, elle eut une ſoif très conſide-

" rable, & cherchoit tous les moyens de la ſatisfaire. Etant

" fille, elle buvoit trois ſeaux d'eau par jour ; étant mariée,

" deux ſeaux lui ſuffirent, juſqu'à ſon premier enfant. Alors

" elle reprit ſa première doſe de trois ſeaux, juſqu'à ſon qua-

" trième

"We accordingly went, on Saturday the

"15th of October, to the woman in queſtion,

"at the Hotel des Arts, Fauxbourg St. Mar-

"tin. Not having met with her at home, we

"went from thence to the place where her huſ-

"band was at work, having previouſly collect-

"ed, from the Porter of the Hotel, ſeveral

"points

"trième enfant; depuis cette epoque, elle n'en boit plus

"que deux dans les 24 heures. Lorſqu'elle eſt malade, elle

"n'a plus la même ſoif; & lorſqu'elle ne boit point autant

"qu'elle le deſire, elle ſe porte mal.

"Lorſqu'elle eſt en couche elle à beaucoup plus ſoif qu'à

"l'ordinaire.

"Elle n'a pas plus ſoif en été qu'en hyver.

"Les choſes ſalées, qu'elle n'aime pas à manger, ne l'al-

"tèrent pas plus que les autres alimens.

"Sa ſoif ſe fait ſentir par une défaillance d'eſtomac, ſem-

"blable à celle que l'on eprouve lorſqu'on a faim. Elle a la

"bouche pateuſe, & ne pourroit, dit-elle, avaler un morceau

"de pain.

"Lorſqu'elle a bû elle ſent vers la region de l'eſtomac un

"froid aſſez conſidérable, qui la fait friſſonner pendant quel-

"que temps; ce qui l'oblige d'être continuellement auprès

"du feu, pour peu qu'il faſſe froid.

"Cette femme a la lèvre inférieure aſſez groſſe & couverte

"de croutes. Cette lèvre lui cuit & lui elance beaucoup,

"ſurtout en été. Elle eſt ſujette à avoir des hemorrhoides qui

"ne fluent pas; alors elle n'a plus mal à la lèvre.

"Elle

" points of information which agreed with
" what had already been told to us. We found
" the woman with a pitcher of water by her
" ſide ; and a day being appointed for the pur-

" Elle a eu onze enfans en dix couches. C'eſt depuis ſon

" premier enfant qu'elle a des hemorrhoides.

" De tous ſes enfans il ne lui en eſt reſté que deux ; preſ-

" que tous ceux qu'elle a nourri ont été ſujets à différentes

" maladies. Son ainé encore exiſtant à une maladie de la

" peau, ſemblable à la gale, mais qui n'eſt cependant pas con-

" tagieuſe. Le plus jeune, qu'elle n'a nourri qu'un mois,

" jouit d'une très bonne ſanté.

" Cette fémme eſt la ſeule de ſa famille qui ait une auſſi

" grande ſoif.

" Elle ſue aſſez, & urine en proportion de ce qu'elle boit.

" Elle ne crache point.

" Elle ne prend ni caffé, ni vin, ni liqueurs ſpiritueuſes.

" Elle nous a dit qu'elle mangeoit beaucoup, ce que nous
" n'avons cependant pas remarqué.

" Cette femme a bû devant nous, pendant dix heures quelle

" eſt reſtée avec nous, quatorze pintes d'eau ; ce qui peut

" produire environ vingt-huit livres. Elle nous a dit qu'elle

" ſe relevoit la nuit toutes les heures & demie pour boire ; ce

" qui fait aſſez exactement la voie d'eau que cette femme pré-

" tend conſommer dans 24 heures.

" Elle a rendû dix pintes d'urine peu colorée.

" M. M. Bonnard, Lair, & Robilliard, membres de la
" Société Philomatique, ont vû cette femme avec nous pen-
" dant une aſſez grande partie de la journée."

" poſe,

" pofe, it was fettled that fhe fhould come and
" pafs the whole of it with us.

" We met accordingly on Monday the 17th
" of October, 1791, and received from this
" woman the following particulars:

" Catherine Bonfergent, wife of James Fery,
" a cobler, now living in the Hotel des Arts,
" Fauxbourg St. Martin, parifh of St. Lau-
" rence, at Paris, is forty years old, and was
" born at Senlis.

" She is very fair; her fkin is fine, but frec-
" kled. She is rather lean than fat, and feems
" to be of a bilious temperament. Her arms
" are leaner than the reft of her body.

" At the time fhe was weaned fhe was placed

" with her grandmother, who, drinking a good

" deal of wine, made her alfo drink it. When

" fhe returned home to her mother fhe vomited

" up every thing fhe took. What fhe vomited

" was of a black colour.

" From her earlieft infancy fhe had a very
" confiderable thirft, and fought every means
" of fatisfying it. While fhe was fingle fhe
" drank three pailfuls of water a day; after fhe
" was married two pailfuls were fufficient for
" her till fhe was delivered of her firft child;
" fhe then returned to her former quantity of

" three

" three pailfuls, and continued it till after the " birth of her fourth child. Since that period " ſhe has drank only two pailfuls in the four " and twenty hours. When ſhe is ſick ſhe has " no longer the ſame thirſt, and when ſhe does " not drink as much as ſhe deſires ſhe is ill.

" When ſhe lays in ſhe has much more thirſt " than uſual.

" She has not more thirſt in ſummer than in " winter.

" Salted meats ſhe is not fond of eating, but " they do not render her more thirſty than " other aliments.

" Her thirſt occaſions a ſenſation at the ſto-" mach ſimilar to that which is excited by hun-" ger. Her mouth is clammy, and ſhe is un-" able, ſhe ſays, to ſwallow a bit of bread.

" When ſhe has drank ſhe feels about the " region of the ſtomach a pretty conſiderable " coldneſs, which occaſions her to ſhiver for " ſome time, and obliges her to be conſtantly " near the fire whenever the weather happens " to be at all cold.

" This woman has the lower lip rather thick, " and covered with ſcabs. This lip ſmarts, " and at times is very painful to her, eſpecially " in ſummer. She is ſubject to the blind piles,

" and

"and when these take place the complaint in

"her lip ceases.

"She has had eleven children in ten lyings

"in. It is since the birth of her first child

"that she has been subject to the piles.

"Of all her children there remain only two.

"Almost all of those she has suckled have been

"subject to different diseases. Her eldest, who

"is still living, has a disease of the skin similar

"to the itch, but which is not infectious. Her

"youngest child, which she has suckled only a

"month, is in very good health.

"This woman is the only one of her family

"who has so great a thirst.

"She perspires sufficiently, and her urine is

"in proportion to what she drinks.

"She does not spit.

"She drinks neither wine, nor coffee, nor
"spirituous liquors.

"She told us that she ate a great deal, but

"we did not observe this while she was with

"us.

"This woman drank, in our presence, du-

"ring the space of ten hours which she remain-

"ed with us, fourteen quarts (or Paris pints)

"of water, which must be equal to about

"twenty-eight pounds. She assured us that in

" the night time ſhe riſes every hour and a half

" to drink, and this will be found to make

" pretty exactly the load, or two pailfuls, of

" water, which this woman aſſerts that ſhe

" drinks in four and twenty hours.

" She voided ten quarts of urine that was

" nearly colourleſs.

" M. M. Bonnard, Lair, and Robilliard,
" members of the Philomatical Society, ob-
" ſerved, with us, this woman during a conſi-
" derable part of the day."

While the preceding account was preparing for the preſs the following paragraph appeared in the Lincoln Mercury of Friday, December 9, 1791:

" However extraordinary the following cir-

" cumſtance may appear, it may be depended

" on as fact:—A man who lives with Mr.

" John Julyan, of Woodſtone, near Peterbo-

" rough, is afflicted with ſuch an immoderate

" degree of thirſt, as obliges him to drink

" the aſtoniſhing quantity of three gallons of

" water a night, and one gallon a day; and

" what makes this appear ſtill more extraordi-

" nary,

" nary, he has continued this practice twenty-

" three years."

The facts described in this paragraph bore too ftriking a refemblance to thofe he had juft before received from Paris not to excite in the Editor a wifh to fee the cafe more fully and fatisfactorily inveftigated. This has fince been done through the kind offices of Sir Jofeph Banks, Bart., who being acquainted with a gentleman in the neighbourhood of the patient, on whofe accuracy he knew he could depend, had the goodnefs to tranfmit to him fome queries from the Editor relative to this fubject, with a requeft that he would engage in the inquiry.

In confequence of this requeft Mr. Maxwell, the gentleman alluded to, fent for the man to his houfe, where he remained a whole night, and was carefully attended to. The refult of this inveftigation, which fufficiently eftablifhes the truth of the facts, we fhall here give in Mr. Maxwell's own words.

*Extract of a Letter from Mr.* George Maxwell *to Dr.* Simmons, *dated Fletton Lodge, near Peterborough, December* 18, 1791.

" With respect to the Water Drinker, who " is the subject of your inquiry, and who lives " at Stanground, near Woodstone, though he " works at the latter place, it happens that Mr. " Beal, the person who now looks after my " farm, employed him as a thrasher more than " twenty years ago. His account of this man " is, that he always drank the quantity he is " now said to do, or at least was at that time " reputed to drink it.

" As he resided three or four miles from " Mr. Beal's habitation, the latter used to make " up a bed for him in his house, and Mr. " Beal observed that at night he always took " a bucketful of water up stairs with him.

" I have a labourer likewise who has worked " with him, and who says that in mowing time " this man always takes four quarts of water " out with him from a pump in the village, " besides two quarts of beer.

" These

" These accounts being sufficiently satisfac-

" tory as to his not being an impostor, I have

" sent for him, and put to him your queries,

" which I shall here set down, together with

" his answers to each :

Q. 1st. " His name, age, occupation, habit

" of body, and general state of health ?

A. " William Read ; in the fifty-first year of

" his age ; a labourer ; never costive ; gene-

" rally in good health.

Q. 2d. " Whether his thirst is natural, or a

" consequence of disease, and if so, at what

" period of life it first shewed itself ?

A. " Not natural, but came on after an ague
" and fever, which confined him a whole win-
" ter, twenty-four years ago.

Q. 3d. " Whether his thirst is constant and

" uniform, the same in summer as in winter,

" or only occasional, and varying in degree ?

A. " Always the same, when he is well.

Q. 4th. " Whether he drinks any other li-

" quor besides water ?

A. " Has no objection to other liquors, but

" can seldom get any.

Q. 5th. " How much does he usually take at

" a draught, and how often does he repeat it ?

A. " A quart at a time, and repeats it sixteen

"or eighteen times in the courſe of a day and

"night.

Q. 6th. "Whether his thirſt is diminiſhed or

"increaſed when his general health happens to

"be affected?

A. "When his health happens to be affected

"he drinks but little; nothing like ſo much as

"the uſual quantity.

Q. 7th. "What is the ſtate of his tongue and

"fauces with reſpect to dryneſs, moiſture, &c.?

A. "No appearance of dryneſs.

Q. 8th. "What quantity of urine does he

"void, and what is the ſtate of it?

A. "He makes water almoſt every time he

"drinks, and as much upon the whole as he

"drinks. He knows nothing of the ſtate of

"it.

Q. 9th. "Does he perſpire much or little?

A. "Very much when he works, but not at

"all in the night.

Q. 10th. "What is the general ſtate of his

"bowels?

A. "No purging, nor any pain in his bowels.

Q. 11th. "Is he the only one of his family

"who has been remarkable for this exceſſive

"thirſt?

A. "Yes.

"The

"The man adds, that he has consulted seve-

"ral medical gentlemen about his complaint,

"but has not been able to get any thing that

"could, in the least, relieve him.

"On Sunday the 18th instant, at two o'clock,

"he ate a hearty dinner of roast beef with my

"servants, and drank a quart or more of beer.

"Contrary to his promise, he went home as

"soon as dinner was over, but returned about

"five, when I ordered him into the room where

"I was sitting, and he drank a quart of water

"at a draught and very greedily. He said he

"had drank three times whilst absent.

"At eight o'clock he supped, and drank a

"quart of small beer.

"At nine o'clock he went to bed.

"Mr. Beal promised to watch him all night.

"At half past nine I went over to Mr. Beal's

"to settle the plan of management, his house

"being at a little distance from mine. It was

"agreed that no water should be left in Read's

"bed room, but that it should be set ready in a

"room below to be carried to him at a quart at

"a time in the night.

"The next morning (Monday), at eight
"o'clock, I found him at breakfast. Mr. Beal

"informed me that he had carried him the "water himself, and that

"At ten o'clock (the night before) he had "drank a quart;

"At eleven o'clock, another quart;

"At twelve o'clock, another quart;

"At near three (Monday morning), another "quart; (all of which he drank greedily; "each at a single draught);

"Between four and five o'clock, another "quart, except a little left in the mug.

"At near six another quart was carried to "him, but of this he left about half.

"A servant boy who slept with him says he "drank the remainder of the last quart after "Mr. Beal left him.

"The patient himself says he drank a seventh "quart as soon as he got up, whilst Mr. Beal "was employed in the yard.

"I found him, as I just now mentioned, at "breakfast, in the kitchen, eating heartily of "milk with bread crumbed in it. He ob- "served to me that he prefers milk to cold "meat or any thing else; that he was not more "thirsty last night than usual, and thinks he "usually drinks as much every night, but that "never having had his liquor measured to him

"before, he could not ſpeak with certainty in

"the account he gave.

"I examined the water made by him in the

"night. There appeared to be between five

"and ſix quarts of it, and it ſhewed no appea-

"rance of ſediment.

"At nine o'clock the man had finiſhed his

"breakfaſt, having ate a quart of milk and

"bread, and ſome cold meat after it, and

"drank two quarts of ſmall beer, except about

"a gill which we found left in the bottom of

"the laſt jug.

"The facts being thus aſcertained without a

"poſſibility of doubt, I did not think it ne-

"ceſſary to detain him any longer; and for

"my own part I believe all that he ſays on the

